# Difference in spectral power density of sleep electroencephalography in individuals with or without insomnia

**DOI:** 10.1192/j.eurpsy.2022.332

**Published:** 2022-09-01

**Authors:** J.M. Kang, S.-E. Cho, S.-G. Kang

**Affiliations:** Gachon University Gil Medical Center, College of Medicine, Department Of Psychiatry, Incheon, Korea, Republic of

**Keywords:** Insomnia, spectral power density, beta power, qEEG

## Abstract

**Introduction:**

Power spectral analysis is the most common method of quantitative electroencephalogram (qEEG) techniques and enables investigation of the microstructure of insomnia. Previous spectral analysis studies on insomnia have shown inconsistent results due to their heterogeneity and small sample sizes.

**Objectives:**

We compared the difference of electroencephalogram (EEG) spectral power during sleep among participants without insomnia, insomniacs with no hypnotic use, hypnotic users with no insomnia complaints, and hypnotic users with insomnia complaints.

**Methods:**

We used the Sleep Heart Health Study data, which is large sample size and has good quality control. The fast Fourier transformation was used to calculate the EEG power spectrum for total sleep duration within contiguous 30-second epochs of sleep. For 1,985 participants, EEG spectral power was compared among the groups while adjusting for potential confounding factors that could affect sleep EEG.

**Results:**

The power spectra during total sleep differed significantly among the groups in all frequency bands (p corr < 0.001). We found that quantitative EEG spectral power in the beta and sigma bands of total sleep differed (p corr < 0.001) between participants without insomnia and hypnotic users with insomnia complaints after controlling for potential confounders. The higher beta and sigma power were found in the hypnotic users with insomnia complaints than in the non-insomnia participants.

**Conclusions:**

This study suggests differences in the microstructures of polysomnography-derived sleep EEG between the insomnia groups.

**Disclosure:**

No significant relationships.

